# Comparison between Low-Level and High-Intensity Laser Therapy as an Adjunctive Treatment for Knee Osteoarthritis: A Randomized, Double-Blind Clinical Trial

**DOI:** 10.3390/life13071519

**Published:** 2023-07-06

**Authors:** Mohd Azzuan Ahmad, Mageswari Moganan, Mohamad Shariff A Hamid, Norhuda Sulaiman, Ushantini Moorthy, Nazirah Hasnan, Ashril Yusof

**Affiliations:** 1 Physiotherapy Programme, Centre for Rehabilitation and Special Needs Studies, Faculty of Health Sciences, Universiti Kebangsaan Malaysia, Kuala Lumpur 50300, Malaysia; azzuanahmad@ukm.edu.my; 2 Faculty of Sports and Exercise Science, Universiti Malaya, Kuala Lumpur 50603, Malaysia; magesmogan@gmail.com; 3 Sports Medicine Unit, Faculty of Medicine, Universiti Malaya Medical Centre, Kuala Lumpur 59100, Malaysia; ayip@um.edu.my (M.S.A.H.); norhudas@ummc.edu.my (N.S.); ushantini@ummc.edu.my (U.M.); 4 Department of Rehabilitation Medicine, Faculty of Medicine, Universiti Malaya, Kuala Lumpur 59100, Malaysia; nazirah@um.edu.my

**Keywords:** high-intensity laser, knee osteoarthritis, low-level laser, pain, photobiomodulation, rehabilitation

## Abstract

Background: Low-level (LLLT) and high-intensity laser therapy (HILT) can be beneficial additions to knee osteoarthritis (KOA) rehabilitation exercises; however, it is still being determined which electrophysical agent is more effective. Aim: To compare the effects of LLLT and HILT as adjuncts to rehabilitation exercises (LL + EX and HL + EX) on clinical outcomes in KOA. Methods: Thirty-four adults with mild-to-moderate KOA were randomly allocated to either LL + EX or HL + EX (*n* = 17 each). Both groups underwent their respective intervention weekly for twelve weeks: LL + EX (400 mW, 830 nm, 10 to 12 J/cm^2^, and 400 J per session) or HL + EX (5 W, 1064 nm, 19 to 150 J/cm^2^, and 3190 J per session). The laser probe was placed vertically in contact with the knee and moved in a slow-scan manner on the antero-medial/lateral sides of the knee joint. Participants’ Knee Injury and Osteoarthritis Outcome Score (KOOS), Numerical Pain Rating Scale (NPRS), active knee flexion, and Timed Up-and-Go test (TUG) were assessed. Results: Post intervention, both groups showed improvements in their KOOS, NPRS, active knee flexion, and TUG scores compared to baseline (*p* < 0.01). The mean difference of change in KOOS, NPRS, and active knee flexion scores for the HL + EX group surpassed the minimal clinically important difference threshold. In contrast, the LL + EX group only demonstrated clinical significance for the NPRS scores. Conclusions: Incorporating HILT as an adjunct to usual KOA rehabilitation led to significantly higher improvements in pain, physical function, and knee-related disability compared to LLLT applied in scanning mode.

## 1. Introduction

Knee osteoarthritis (KOA) is a global public health concern and one of the leading causes of physical impairment and disability worldwide [1,2]. As there are currently no single disease-modifying interventions for KOA [3], conservative treatments such as pharmacological therapy and rehabilitation exercises have often been accompanied by the use of electrophysical agents to optimize treatment outcomes [4]. These include low-level (LLLT) [5,6] and high-intensity laser therapy (HILT) [7,8], therapeutic ultrasound, and transcutaneous electrical nerve stimulation [9]. In recent years, photobiomodulation therapy specifically, LLLT and HILT have emerged as the most promising modalities [4,8,10], as both variants can reduce pain and inflammation [6,7], augment tissue repair [11,12], increase blood circulation [13], and improve physical function and performance [6,7]. Since previous trials have predominantly evaluated either LLLT or HILT without comparing them directly [14,15], it remains unclear which electrophysical agent is more effective in treating KOA [6,8,16].

Photobiomodulation therapy is a non-invasive electrophysical agent that utilizes therapeutic doses of light to target injured or dysfunctional tissue, activating photo-biological mechanisms for pain relief and tissue healing [11,12]. It is theorized that when using laser wavelengths of 660 nm and above for photobiomodulation, the laser energy (photons) is absorbed by the tissue and cells, initiating cellular mitochondrial oxidative reactions that yield adenosine triphosphate (essential for optimal cell metabolism and healing), modulate low-level reactive oxygen species, and release nitric oxide as a potent vasodilator to reduce pain and inflammation [11,12]. Low-level laser therapy (<500 mW) was first investigated with regard to KOA by Gur et al. (2003) [17]. Later, it was postulated that high-intensity laser therapy, which delivers a higher energy output (greater than 500 mW per laser diode), has the potential to penetrate deeper tissue and induce superficial hyperthermia, thereby harnessing photothermic effects [11]. Additionally, HILT, with wavelengths over 1064 nm, has been demonstrated to target nerve endings directly, providing immediate pain relief [8,11]. Due to its greater anti-inflammatory, bio-stimulation, and photothermic advantages, HILT is considered by some to be more promising than LLLT for the treatment of KOA [4,11,16]. Moreover, single-treatment clinical trials (indirect comparison) and meta-analysis suggest that HILT (1064 nm; 1250 to 3000 J per session) is more effective than LLLT (640 to 905 nm; 3 to 1250 J per session) [16]. However, a direct comparison between LLLT and HILT on KOA outcomes is lacking to support this supposition.

Kheshie et al. (2014) [18] performed one of the few studies to date that compared the effects of HILT and LLLT as an adjunct to KOA rehabilitation [18]. A similar dosage (1250 J) was administered for both the HILT and LLLT treatment groups. It is worth noting that in the Kheshie et al. (2014) [18] study, a higher dose of LLLT (1250 J/session) was used compared to other related studies [19,20,21]. Nonetheless, the study found that HILT was better than LLLT in improving KOA outcomes [18], suggesting that power levels and pulse duration play important roles. HILT uses lasers with higher power and shorter pulse durations than LLLT, allowing it to penetrate deeper and create a more significant biological effect [11]. Therefore, it is believed that even with the same dosage as HILT, a high dose of LLLT may not translate into better outcomes if the laser’s power is unable to penetrate deep enough [11]. Nevertheless, the study’s small sample size, which only included male participants, and the reliance solely on patient-reported outcomes (VAS and WOMAC) limit the generalizability of its findings. The sampling limitations are particularly significant considering the fact that KOA is more prevalent in females [1]. Moreover, patient-reported outcome measures may be challenged due to the potential for subjective and biased interpretations [22]. Thus, a randomized trial using robust research methods is warranted to distinguish the clinical effects of LLLT and HILT.

To sum up, current clinical evidence for comparing the effects of LLLT and HILT in treating KOA is limited due to methodological issues such as the use of single-treatment clinical trials and single-sex participant samples, limited reliability and validity of outcome measures, and the application of meta-analysis. As such, this study was designed to compare the clinical effects of LLLT and HILT as adjunctive treatment alongside rehabilitation exercises on pain, function, and disability levels in adults with mild-to-moderate KOA. The findings of this study could provide a valid justification for including laser therapies as a mainstream management option for KOA and enable health professionals to select the most efficient electrophysical agent (LLLT or HILT) for optimal outcomes. It was hypothesized that HILT would provide better clinical outcomes than LLLT as an adjunctive treatment for KOA.

## 2. Materials and Methods

### 2.1. Study Design

This study was a randomized, double-blind (participants and outcome assessor), parallel-group clinical trial.

### 2.2. Ethical Approval and Registration

The study protocol was approved by the Medical Research Ethics Committee of Universiti Malaya Medical Centre (MREC UMMC ID: 2020102-9129) in accordance with the Declaration of Helsinki (1975). The study protocol has been prospectively registered with the Australian New Zealand Clinical Trials Registry (ACTRN12621001694808) and the National Medical Research Register of the Ministry of Health Malaysia (NMRR-21-86-58301).

### 2.3. Study Population and Setting

Patients with symptomatic mild-to-moderate KOA were screened and recruited from the Sports Medicine Clinic of UMMC. Eligible KOA patients were invited to participate in this study based on the following inclusion and exclusion criteria.

Inclusion: Aged 18 years and above, diagnosed with unilateral or bilateral KOA based on the American College of Rheumatology criteria, categorized as mild-to-moderate KOA according to the Kellgren-Lawrence radiographic classification, and presented with knee pain; if both knees were affected, the knee with worse symptoms was included in the outcome assessment.

Exclusion: (i) Recent intra-articular knee injection; less than six months, (ii) any other pathological conditions such as rheumatic disease, hip or knee joint replacements, congenital dysplasia, septic arthritis, ligament or meniscus injury, plica syndrome, and Baker’s cyst, or (iii) those with comorbidities that would prevent participation in the intervention or physical evaluation.

### 2.4. Sample Size Calculation

The sample size was estimated using the G*Power software version 3.1.9.7. The calculation was based on a minimal clinically important difference (MCID) of 11 points for the Knee Injury and Osteoarthritis Outcome Score (KOOS) as the primary outcome [23], assuming a pre-specified power of 90%, an effect size of 0.3, an alpha level of 5%, and a possible dropout rate of 15% [21,24]. Thus, the required total sample was determined to be 34, with 17 participants per group.

### 2.5. Procedures

Patients with mild-to-moderate KOA were screened and recruited from the Sports Medicine Clinic of UMMC through simple random sampling, provided they fulfilled the inclusion and exclusion criteria. Eligible participants received verbal and written information about the study protocol and signed consent forms before participating. A total of 34 participants were randomly assigned in a 1:1 ratio to one of two intervention groups (LL + EX or HL + EX) using a computer-generated randomization table. The allocation concealment was achieved through sealed opaque envelopes that contained information about the treatment group. Screening, recruitment, and randomization were carried out by a researcher who was not involved in intervention implementation or outcome evaluation.

Patients in both groups received personalized knee rehabilitation exercises (usual physiotherapy treatment) weekly for twelve weeks. The knee rehabilitation exercises were structured as individualized one-to-one sessions conducted by qualified physiotherapists from the Sports Medicine Clinic of UMMC who were unaware of the participants’ group allocations. Each session typically lasted for one hour. The usual physiotherapy treatment was prescribed and performed, tailored to each individual’s needs, and followed recommended KOA treatment guidelines [25,26]. The personalized knee rehabilitation program consisted of a wide range of therapeutic exercises, tailored to the individual’s needs. These exercises included stretching, strengthening, balance training, proprioception exercises, and functional movements, as well as manual therapies such as soft tissue mobilization and non-thrust manipulation techniques like oscillatory patellofemoral and tibiofemoral mobilization [25,26]. Examples of the KOA-specific exercises included calf stretching, straight leg raise, static quadriceps, terminal knee extension, and sit-to-stand exercises [25,26,27,28].

In addition to the standardized knee rehabilitation exercises, all participants received either HILT or LLLT based on their group allocation. Laser protocols were adapted based on previous studies [18,29,30,31]. The laser treatment procedures were the same for both groups, as follows: (i) patient position: supine with their knee flexed at 30° to allow for laser irradiation to reach the joint surfaces [18,32]; (ii) treatment area: antero-medial and antero-lateral sides of the knee joint which cover approximately 20 cm^2^ per knee [18,32]; (iii) application: the laser probe was placed vertically against the knee and slowly moved in a longitudinal and perpendicular direction; and (iv) treatment time: 15 min (5 min of pulse mode and 10 min of continuous mode) of laser treatment per knee. Additionally, participant safety was prioritized throughout the study, and careful monitoring of potential adverse effects was conducted during the laser treatment and subsequent follow-up periods. Participants were instructed to promptly report any pain, discomfort, or adverse effects associated with the laser therapy. The interventions for both groups were administered once a week for 12 consecutive weeks. Each group received the following laser parameters:

Group LL + EX (*n* = 17): Participants in this group received LLLT in conjunction with their knee rehabilitation exercises. The LLLT was administered using the BTL-5825SL (BTL Int) with a wavelength of 830 nm, peak power output of 400 mW, an energy density of 10 to 12 J/cm^2^, and a total energy of 400 J (100 J pulsed and 300 J continuous mode) during each session.

Group HL + EX (*n* = 17): Participants in this group received HILT in addition to their knee rehabilitation exercises. The HILT was administered using the BTL 6000 HIL (BTL Int) 12 Watt, with a wavelength of 1064 nm, a power output of 5 W, an energy density of 19 to 150 J/cm^2^, and a total energy of 3190 J (190 J pulsed and 3000 J continuous mode) during each session.

### 2.6. Standardization and Blinding

To ensure the blinding of the intervention, all laser treatment sessions (LLLT and HILT) were identical between the two groups (including laser preparation, procedures, and the duration of treatment). In this study, both laser treatments (LLLT and HILT) were consistently administered by one of the authors, who was not involved in the outcome assessment. It is important to note that the author who performed the laser treatment underwent specialized training to ensure proficiency. Meanwhile, all outcome measures were performed by a single assessor who was blinded to group allocation.

### 2.7. Outcome Measures

The outcomes of this study were assessed using the Knee Injury and Osteoarthritis Outcome Score (KOOS) for knee-related disability, the Numerical Rating Pain Scale (NPRS) for pain, active knee flexion for physical function, and the Timed Up-and-Go test (TUG) for functional performance. All outcomes were assessed by the same assessor at baseline (Week 0) and immediately after completion of the intervention (Week 12).

The KOOS is a self-administered questionnaire used to evaluate participants’ opinions regarding their knee problems through five specific subscales: pain, symptoms, activities of daily living (ADL), sport and recreation, and quality of life (QOL) [33]. The English and Malay versions of the KOOS were employed in this study, and each item is rated on a five-point Likert scale (0 to 4). The total score for each subscale is transformed into a percentage, with 0% indicating severe knee problems or high knee-related disability and 100% indicating no knee problems or knee-related disability [34]. Validity studies have consistently demonstrated a moderate-to-high correlation between KOOS subscales and WOMAC subscales [33], with interclass correlation coefficients ranging from 0.91 to 0.99 [35] and Cronbach’s alpha values ranging from 0.84 to 0.91 [34], indicating excellent test-retest reliability. Additionally, a previous study reported that the Malay version of the KOOS questionnaire demonstrated an excellent degree of goodness of fit and was found to be a valid and reliable tool for assessing Malaysian adults with KOA [36].

Meanwhile, the NPRS is a simple, widely used, unidimensional measure of pain, in which the participants select a whole number (0 to 10) that best reflects their pain intensity, ranging from 0 (no pain) to 10 (the worst imaginable pain) [37]. It has excellent test-retest reliability, with an intraclass correlation coefficient, a standard measurement error, and a minimal detectable change of 0.95, 0.48, and 1.33, respectively [38]. Active knee flexion was measured using a universal goniometer (2 × 25 cm). It has been found that the universal goniometric assessment of the knee joint are both valid and reliable, with high inter- (>0.99) and intra-rater (>0.98) reliabilities [39].

The TUG test is a reliable and valid method for measuring functional mobility, balance, and fall risk [40,41]. It is cost-effective and time-efficient, with an intra-rater reliability of 0.97 and an inter-rater reliability of 0.96 [40]. The construct validity of TUG performance has been correlated with the Berg Balance Scale, the 10 m gait speed test, and the Bartell Index [41]. In this study, the assessor demonstrated the TUG test to the participants. Participants were instructed to sit on a standard armchair, rise from the chair, walk at a safe and comfortable pace to a marker 3 m away (marked by a line and a cone), walk around the cone, and return to the chair to sit down again. They were allowed to use walking aids and hold the armrest to sit and stand during the test. The time taken from the start of movement from the chair to sitting back on the chair was recorded, and a score of more than 14 s indicated low functional mobility performance and a high risk of falls [40].

### 2.8. Statistical Analysis

Data were analyzed using SPSS version 25.0 (SPSS Inc., Chicago, IL, USA) based on the intention-to-treat principle, where all participants were included in the analysis, regardless of their adherence to the intervention. Descriptive statistics and cross-tabulations were used to describe participants’ sociodemographic characteristics at baseline, including age, gender, body mass index, duration of illness, affected side, KOA severity, and use of mobility aid. The baseline comparison of clinical outcomes was performed using a one-way analysis of variance to assess the impact of these factors as dependent variables. The primary aim of the study was to examine the within- and between-group differences in the pre- and post-intervention KOOS, NPRS, active knee flexion, and TUG scores of the LL + EX and HL + EX groups, using analysis of covariance (ANCOVA). The validity and reliability of the ANCOVA were ensured by conducting assumption checks for normal distribution and homogeneity of variance, which were passed. To evaluate the magnitude of the difference between groups, the effect size of each variable was determined using Cohen’s d, with 0.2, 0.5, and 0.8 representing small, medium, and large effects, respectively [24]. Furthermore, the MCID was considered for the KOOS, NPRS, active knee flexion range, and TUG to assess the clinical relevance of the observed changes [23,42,43]. All statistical analyses were conducted using an alpha level of 0.05 for all significance tests.

## 3. Results

### 3.1. Participants’ Characteristics

**Figure 1:**
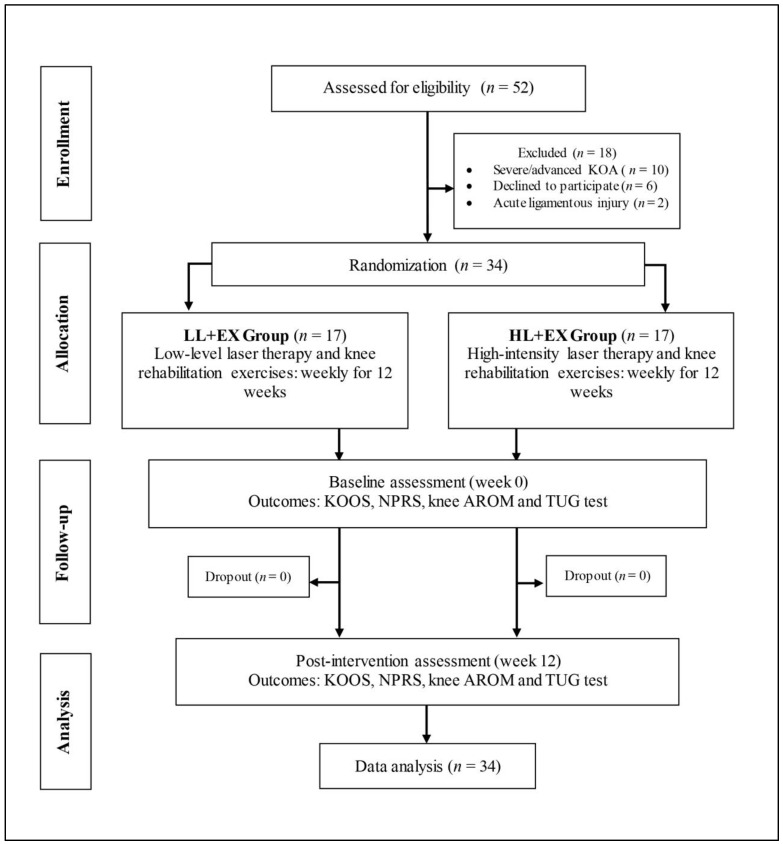
Study flowchart.

Fifty-two patients with symptomatic KOA were screened based on the study inclusion and exclusion criteria for eligibility at the Sports Medicine Clinic of UMMC. Of these, 18 were excluded for the following reasons: (i) categorized as severe/advanced KOA (*n* = 10), (ii) refusal to participate (*n* = 6), and (iii) underlying acute ligamentous knee injuries (*n* = 2). Thirty-four patients (76% female and 24% male) with mild-to-moderate symptomatic KOA agreed to participate in this study and were randomized into one of two study groups (Figure 1): LL + E (*n* = 17; five males and twelve females) and HL + EX (*n* = 17; three males and fourteen females). The mean ± standard deviation (SD) for age, body mass index (BMI), and the duration of KOA in the LL + EX and HL + EX groups were 57.94 ± 10.56 years, 27.57 ± 4.47 kg/m^2^, and 38.35 ± 28.26 months; and 51.18 ± 9.79 years, 30.58 ± 5.43 kg/m^2^, and 39.88 ± 39.11 months, respectively. Additionally, about 79% (*n* = 27) of the participants were identified with bilateral KOA involvement, while 59% (*n* = 20) were identified as having moderate KOA, and 41% (*n* = 14) as having mild KOA, based on the Kellgren-Lawrence classification. Furthermore, 18% (*n* = 6) were using mobility aids for ambulation, mostly single-point walking sticks. The baseline sociodemographic and clinical outcome characteristics of the participants in both groups are summarized in Table 1.

### 3.2. Baseline Comparability Analysis

There were no significant differences in participants’ sociodemographic variables (age, gender, and BMI), disease characteristics (duration of illness, affected side, severity of KOA, and use of mobility aid), or outcome variables (KOOS, NPRS, active knee flexion, and TUG) between the two groups at baseline.

### 3.3. Evaluation of Outcomes

All participants completed the twelve intervention sessions and went through the pre-post assessments, representing an adherence rate of 100%. No adverse effects of laser therapy were reported during the study period. The mean scores of the primary and secondary outcomes with their respective standard deviations at baseline (week 0) and post intervention (week 12) are reported in Table 2.

### 3.4. Changes in KOOS Scores

**Figure 2:**
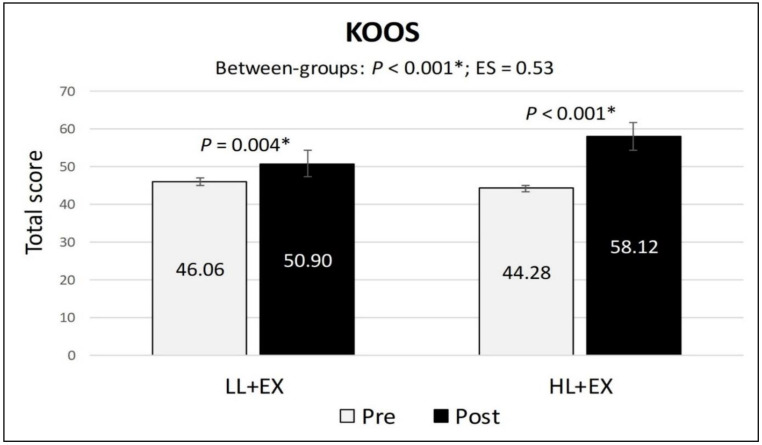
Changes in the KOOS total score pre-post intervention. Abbreviations: ES, effect size; HL + EX, high-intensity laser therapy and exercises; KOOS, knee injury and osteoarthritis outcome score; LL + EX, low-level laser therapy and exercises. Statistically significant, * *p* < 0.05.

In terms of the main outcome (KOOS), participants in both groups showed a statistically significant within-group increase in the KOOS total score following the completion of the treatment (*p* < 0.001), suggesting an amelioration of KOA symptoms and enhanced participation in functional activities, thus reducing the level of knee-related disability. However, the mean differences of change for the KOOS total score were significantly higher in the HL + EX group (MD: 13.84; 95% CI: 9.83 to 17.85; *p* < 0.001) compared to the LL + EX (MD: 4.84; 95% CI: 1.74 to 7.94; *p* = 0.004) (Figure 2). Moreover, the analysis of covariance for all the KOOS subscales scores showed statistically significant mean differences of change between the two groups, favoring HL + EX (*p* < 0.01), with the effect size (Cohen’s d) ranging from 0.14 (small) to 1.04 (large).

### 3.5. Changes in NPRS, Active Knee Flexion, and TUG Scores

**Figure 3:**
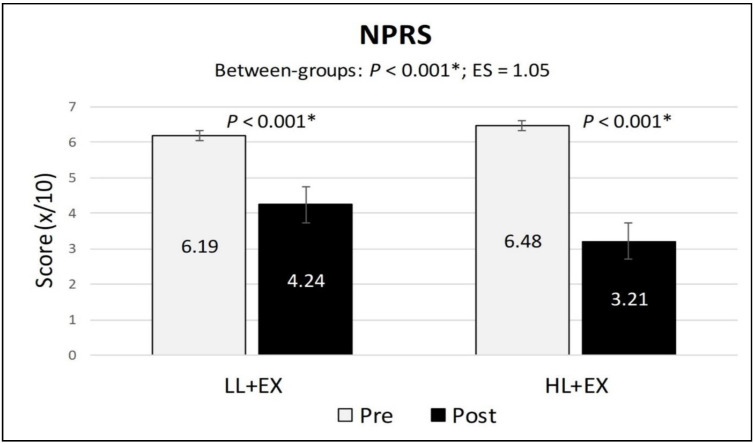
Changes in the NPRS scores, pre and post intervention. Abbreviations: ES, effect size; HL + EX, high-intensity laser therapy and exercises; LL + EX, low-level laser therapy and exercises; NPRS, numerical pain rating scale. Statistically significant, * *p* < 0.05.

**Figure 4:**
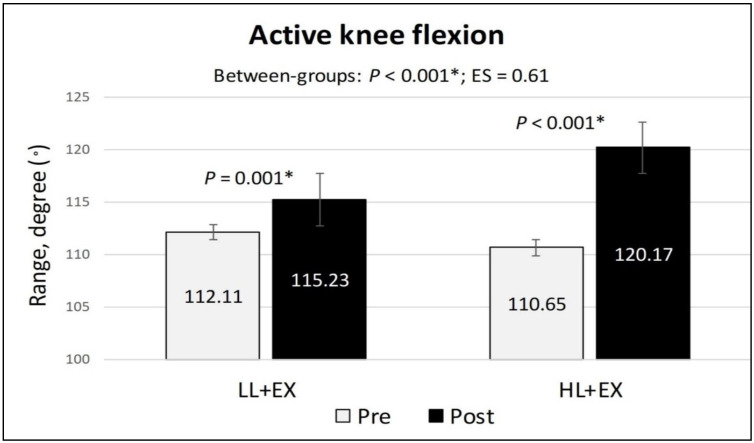
Changes in the active knee flexion scores, pre and post intervention. Abbreviations: ES, effect size; HL + EX, high-intensity laser therapy and exercises; LL + EX, low-level laser therapy and exercises. Statistically significant, * *p* < 0.05.

**Figure 5:**
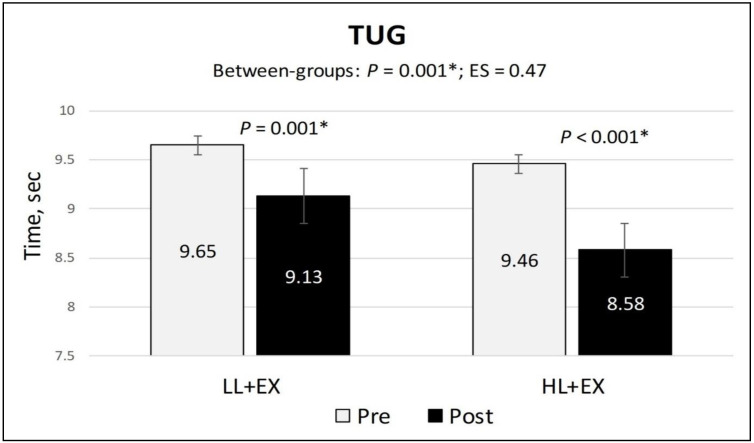
Changes in the TUG scores, pre and post intervention. Abbreviations: ES, effect size; HL + EX, high-intensity laser therapy and exercises; LL + EX, low-level laser therapy and exercises; TUG, timed up-and-go test. Statistically significant, * *p* < 0.05.

The within-group analysis of the secondary outcomes found a statistically significant reduction in the NPRS and TUG scores (indicating a reduction in pain and improvement in functional mobility) and a significant increase in active knee flexion (denoting an improvement in physical function) in both groups compared to baseline (*p* < 0.001). Similarly, the mean differences of change for all the secondary outcomes were higher in the HL + EX group (NPRS (MD: −3.28; 95% CI: −3.78 to −2.76; *p* < 0.001), active knee flexion (MD: 9.53; 95% CI: 5.57 to 13.49; *p* < 0.001), and TUG (MD: −0.88; 95% CI: −1.04 to −0.72; *p* < 0.001)) compared to the LL + EX (NPRS (MD: −1.95; 95% CI: −2.3 to −1.53; *p* < 0.001), active knee flexion (MD: 3.12; 95% CI: 1.56 to 4.68; *p* = 0.001), and TUG (MD: −0.53; 95% CI: −0.64 to −0.41; *p* < 0.001)) (Figure 3, Figure 4 and Figure 5). Between-group analysis of covariance for NPRS, active knee flexion, and TUG found statistically significant higher mean differences of change in the HL + EX group compared to LL + EX both pre and post intervention (*p* < 0.01). Cohen’s d analysis of effect size between the HL + EX and LL + EX groups indicated a large effect for NPRS (d = 1.05), a medium effect for active knee flexion (d = 0.61), and a small effect for TUG (d = 0.47).

### 3.6. Evaluation of Participants’ Blinding Success

Immediately after the last intervention session, all participants were asked to guess whether they had received LLLT or HILT during the last 12 sessions. The results showed that 56.3% of the participants incorrectly guessed their laser treatment, while only 43.7% (47.4% of LL + EX and 52.6% of HL + EX) correctly guessed the type of laser treatment they had received. The Chi-square test analysis revealed no significant difference between the recorded answers, indicating that the blinding of treatment among the participants was successfully maintained.

## 4. Discussion

This study compared the effects of LLLT and HILT as adjunctive treatment to rehabilitation exercises on pain, physical function, and disability levels among patients with mild-to-moderate KOA. The respective laser and exercise interventions for both studied groups (LL + EX and HL + EX) were administered across 12 weekly sessions, and the study outcomes were evaluated at baseline and immediately post intervention. Results showed that both groups exhibited statistically significant reductions in knee pain and disability scores, along with improved physical function and functional mobility compared to baseline; however, the HL + EX group exhibited significantly higher mean differences of change, higher by 50% for NPRS, 20% for KOOS, 6% for active knee flexion, and 3% for TUG relative to the LL + EX group. Importantly, the changes in KOOS and NPRS scores observed in the HL + EX group surpassed the minimal clinically important difference (MCID) threshold, indicating a significant decrease in knee-related disability (MCID of 11.1 for total KOOS score) [23] and pain (MCID of −2.0 change in NPRS score) [43]. On the other hand, only the NPRS scores of the LL + EX group achieved clinical significance, while no clinically significant changes were observed for functional mobility as measured by the TUG test in either laser group. This study’s clinical evidence implies that combining HILT with usual KOA rehabilitation exercises leads to more substantial positive changes in clinical outcomes, specifically in pain and knee-related disability, compared to LLLT.

In this study, the dosages and treatment mode (stationary or scanning) for LLLT (400 mW, 830 nm, 10 to 12 J/cm^2^, and 400 J per session) and HILT (5 W, 1064 nm, 19 to 150 J/cm^2^, and 3190 J per session) were based on pre-set parameters, as stated in the manufacturer’s guideline, and information from previous, related studies [22,31]. It is noted that, Stausholm et al. (2019) in their systematic review suggested, for stationary application, a lower LLLT dosage (4 to 8 J with 785 to 860 nm wavelength per treatment spot, 2–5 times per week) in treating KOA pain [6]. Meanwhile, in this study, a higher dosage of 400 J per session with an 830 nm wavelength was used over the antero-medial and antero-lateral parts of the knee, using scanning mode, covering around 20 treatment spots of 1 cm² each, resulting in an average of 20 J per treatment spot, which is 2.5 times higher than the suggested dosage by Stausholm et al. (2019). Interestingly, our study revealed that employing a higher energy dosage and reducing the treatment frequency (once a week) of LLLT could enhance pain outcomes in individuals with KOA. However, the LLLT dosage used in this study was not excessively high compared to that used by Kheshie et al. (2014) [18], who administered 1250 J per session [19]. Notably, none of the participants who received the LLLT in the present study reported experiencing any adverse effects. In this study, scanning mode was used for both LLLT and HILT, mainly to standardize treatment between groups. Additionally, scanning mode enables larger treatment areas, which is particularly useful for larger body parts like the knee joint. Moreover, with specific consideration of HILT (as it delivers a higher dose of laser energy), scanning mode is preferable as it theoretically allows for deeper penetration, greater stimulation of cellular processes, and better heat dissemination (to avoid skin burn) [18]. Nonetheless, based on the evidence put forth, speculatively, applying LLLT in a stationary manner instead of scanning mode could also produce desirable outcomes since LLLT is a non-thermal laser, and its therapeutic benefits may be enhanced by concentrating the laser energy in treatment spots [11]. Furthermore, the variation in laser wavelengths, as observed between HILT (1064 nm) and LLLT (830 nm), can have significant implications for tissue penetration [44]. Shorter wavelengths, such as 830 nm, are more readily absorbed by superficial tissues and have limited penetration depth [44]. On the other hand, the higher wavelength of 1064 nm used in HILT allows for greater tissue penetration. While the variation in penetration depth between LLLT and HILT in the present study may have potentially benefitted the group receiving HILT, it is crucial to acknowledge that the depths of both PBM have not been definitively established.

This study acknowledged the work of Kheshie et al. (2014) [18] and Delkhosh et al. (2018) [19], who evaluated the effects of HILT and LLLT as adjunctive treatment to KOA rehabilitation exercises. Kheshie et al. (2014) [18] concluded that HL + EX was more effective than LL + EX in reducing pain and knee-related disability levels in patients with mild-to-moderate KOA. However, a few methodological issues may have confounded their findings. Kheshie et al. (2014) [18] administered a homogenized dose of laser treatment of 1250 J per session; therefore, participants’ treatment times varied between LLLT (33 min) and HILT (15 min). The study also employed a single-blind design which could have introduced assessor bias [45]. Additionally, only male patients were recruited [18]; thus, the results may not be generalizable to females, who have a higher representation [1].

Meanwhile, Delkhosh et al. (2018) [19] compared the effects of HL + EX and LL + EX on pain and knee-related disability in 45 female patients with KOA [19]. The study relied on self-reported outcomes (VAS and WOMAC), similar to Kheshie et al. (2014) [18]. The results showed that both treatments had similar effects in reducing pain and knee-related disability measured by pain VAS and WOMAC, respectively [19]. However, the study determined LLLT to be more appropriate due to its lower cost [19]. The article was only available in English as an abstract, with the rest in Persian, making accurate details on the study’s design, methodology (laser intervention), and results inaccessible. Nevertheless, information on the laser intervention was accessed based on the available trial protocol registration details. The study applied ten intervention sessions over two weeks, with five sessions occurring per week [19]. The LL + EX group received a laser output of 30 mW (830 nm wavelength), while the HL + EX group received 3.2 W of power output (910 nm wavelength) [19]. Unfortunately, no information was available on important details such as treatment time, mode and location of laser application, energy density, or total energy delivered per session. Therefore, a comparison between the total laser dosage delivered based on the present study and Delkhosh et al. (2018) [19] cannot be made. However, we believe that our laser treatment delivers a higher total energy compared to the study by Delkhosh et al. (2018) [19], as we applied 12 sessions with power outputs ranging from 400 mW (LLLT) to 4 W (HILT), which may justify our significant findings regarding the difference between the two laser treatments. Hence, a double-blind (participants and outcome assessors) study design involving patients with KOA of both sexes and the combination of self-reported, clinical, and performance-based outcome assessments could provide more reliable and valid results [24].

Based on recent studies, combining laser treatment, i.e., LL + EX [6,20] or HL + EX, with exercises [8,15,30] was more effective in reducing knee pain and stiffness and enhancing physical function among patients with KOA than rehabilitation exercises alone. These improvements were attributed to the synergistic effects of laser technology and therapeutic exercises on tissue repair at the cellular level [11,12]. Specifically, in this study, the higher reduction in knee pain scores in the HL + EX group compared to the LL + EX group can be attributed to the enhanced properties of high-power laser technology. These advantages include (i) a higher energy output than low-level laser therapy [11]; (ii) an anti-inflammatory effect with pain modulation and impact on nerve endings for pain relief [8,11]; and (iii) a scattering mode of laser radiation with therapeutic photo-thermal effects that induce localized muscle relaxation, reducing muscle spasms [13]. Additionally, regarding pain modulation and the suppression of inflammation, HILT was found to induce the release of endorphins and serotonin at the peripheral nerve endings and decrease proinflammatory cytokines and other inflammatory mediators, such as interleukin-1, interleukin-6, prostaglandin, C-reactive protein, and tumor necrosis factor-alpha [46]. Moreover, HILT increases local tissue temperature and blood circulation in knee joints, promoting the exchange of nutrients in cartilage, stimulating tissue regeneration, and reducing pain, oedema, and inflammation [4]. Consequently, these mechanisms lead to better outcomes following HILT than LLLT in relieving KOA pain [15,16].

Meanwhile, based on the assessments of active knee flexion range and functional mobility (TUG), the group receiving HL + EX showed a 9% increase in active knee flexion and TUG, which was better compared to LL + EX (3% for active knee flexion and 6% for TUG). These improvements could be attributed to the established KOA rehabilitation exercises prescribed as their primary treatment, including (i) stretching exercises, which are effective in increasing active joint ROM by developing greater stretch tolerance [27,28]; and (ii) strengthening exercises for the quadriceps and hamstrings muscles, which serve as knee joint dynamic stabilizers, resulting in higher cadence and lower risk of falls [27,47]. Besides the prescribed rehabilitation exercises, better pain management with laser treatment can enhance physical capacity and performance [6,8], especially through HILT [16]. Furthermore, in the present study, HL + EX has been demonstrated to produce a higher reduction in knee-related disability level, as measured using the KOOS (reduction in KOA pain and symptoms, increase in ADL and sports participation, and improvement of QOL), compared to LL + EX. Previous research has suggested that disability in individuals with KOA results from the intricate interactions between knee pain as the primary symptom and a physical function [48,49]. Therefore, it can be expected that reducing knee pain (as evaluated by NPRS) and increasing the knee joint’s range of motion and functional mobility (measured through active knee flexion and TUG) would be reflected through a reduction in the level of knee-related disability [48,49,50]; this was indicated by the amelioration of KOA symptoms and improvement of functional activity participation, as measured by the KOOS.

This double-blind trial was conducted with a heterogeneous sample, and random group allocation was used to assign participants to groups. The baseline comparison revealed no significant differences between the groups in terms of baseline clinical outcomes or sociodemographic factors as potential confounding variables. In addition, all of the participants across both groups completed their respective treatment protocols and pre- and post-treatment assessments. Findings from the multi-modal assessments, including self-reported, clinician-administered, and performance-based evaluations, may provide sufficient evidence to support the integration of HILT in the management of mild-to-moderate KOA. However, some limitations need to be considered. Firstly, we acknowledge that age can potentially influence symptom severity and treatment effectiveness. Although no statistically significant difference in age was observed between the LL + EX and HL + EX groups, it is important to take this factor into account when generalizing the findings. Second, this study acknowledges that HILT tends to produce heat in the skin, which could potentially reveal the group allocation and affect the blinding of the participants; however, we would like to point out that the laser probe was moved slowly to disseminate heat. Additionally, the evaluation of the participants’ blinding success showed no significant difference between the recorded answers, as confirmed by the Chi-square test analysis. This finding suggests that the blinding of treatment among the participants was successfully maintained despite the potential heat generation from HILT.

Apart from that, it is acknowledged that without a sham treatment group, it is difficult to definitively attribute the observed effects solely to the active laser intervention, considering that the placebo effect can contribute to subjective improvements reported by participants [22]. Additionally, the biphasic effect is also commonly observed in photobiomodulation studies [51], where better outcomes may be detected at a relatively higher (wavelength, intensity, and treatment duration) compared to a lower dosage [11,51]. Furthermore, the generalization of this study is limited to a 3-month intervention period, as long-term follow-up studies, such as the one conducted by Stausholm et al. (2022), have provided valuable insights into the implications of photobiomodulation (PBM) in KOA pain and functional management [19,29]. The long-term effectiveness of laser treatment is critical as KOA is a chronic condition that necessitates lifelong management [1]. Therefore, it is recommended that any future comparative study incorporates a sham treatment group and employs follow-up periods to evaluate the long-term implications.

## 5. Conclusions

This study found that combining LLLT or HILT with usual KOA rehabilitation exercises resulted in statistically significant improvements in knee pain, knee-related disability, physical function, and functional mobility. Interestingly, the results also indicate that combining HILT with exercises produces greater positive changes than using LLLT as an adjunctive therapy to rehabilitation exercises in patients with KOA; specifically, the improvements in knee pain, physical function, and knee-related disability surpassed clinical relevance capacity. Thus, this study supports the consideration of HILT as a more effective treatment option than LLLT in the management of KOA when the interventions are applied in scanning mode. These findings contribute to the growing body of evidence supporting the use of laser therapy, particularly HILT, in conjunction with rehabilitation exercises as a viable treatment approach for KOA.

## Figures and Tables

**Table 1:** The demographic characteristics and clinical outcome scores of the participants at baseline.

Variables	LL + EX (*n* = 17)	HL + EX (*n* = 17)	*p*-Value
Age (years)	57.94 (10.56)	51.18 (9.79)	0.062
Gender (male/female)	5/12	3/14	0.344
Body mass index (kg/m²)	27.57 (4.47)	30.58 (5.43)	0.088
KOA duration (months)	38.35 (28.26)	39.88 (39.11)	0.897
Affected side (uni/bilateral)	5/12	2/15	0.199
K-L grade (I/II/III)	0/5/12	0/9/8	0.142
Mobility aid (yes/no)	14/3	14/3	0.672
KOOS (total score)	46.06 (9.47)	44.28 (12.41)	0.642
NPRS (score)	6.19 (1.45)	6.48 (1.21)	0.537
Active knee flexion (degree)	112.11 (11.87)	110.65 (9.73)	0.697
TUG (time, s)	9.65 (1.51)	9.46 (0.75)	0.639

Abbreviations: HL + EX, high-intensity laser therapy and exercises; KOA, knee osteoarthritis; KOOS, knee injury and osteoarthritis outcome score; K-L, Kellgren-Lawrence classification; LL + EX, low-level laser therapy and exercises; NPRS, numerical pain rating scale; TUG, timed up-and-go test. Note: Values are reported as means and standard deviations, with the exception of gender, affected side, severity, and the use of mobility aids, which are presented as frequencies. Categorical variables were analyzed using cross-tabulations and the Chi-square test, while one-way ANOVA was utilized to analyze continuous data.

**Table 2:** Changes in the KOOS, NPRS, active knee flexion, and TUG scores following twelve sessions of intervention for both groups.

Outcomes		LL + EX (*n* = 17)	HL + EX (*n* = 17)	Between-Groups Analysis of Covariance	
		Mean (SD)	Mean (SD)	*p*-Value	Effect Size
KOOS (Total score)	Pre	46.06 (9.47)	44.28 (12.41)	*p* = 0.001 *	0.53
	Post	50.90 (13.92)	58.12 (13.25)		
	MD; 95% CI; *p*	4.84; 1.74 to 7.94; *p* = 0.004 *	13.84; 9.83 to 17.85; *p* < 0.001 *		
Symptoms	Pre	10.87 (3.83)	11.75 (2.65)	*p* = 0.002 *	0.14
	Post	9.73 (3.83)	8.32 (1.60)		
	MD; 95% CI; *p*	−1.12; −1.94 to −0.32; *p* = 0.009 *	−3.43; −4.61 to −2.25; *p* < 0.001 *		
Pain	Pre	18.32 (5.70)	20.14 (6.38)	*p* < 0.001 *	1.04
	Post	15.86 (5.50)	11.09 (3.39)		
	MD; 95% CI; *p*	−2.46; −3.97 to −0.94; *p* = 0.003 *	−9.05; −11.68 to −6.40; *p* < 0.001 *		
ADL	Pre	36.73 (9.28)	32.51 (11.48)	*p* = 0.014 *	0.26
	Post	39.34 (8.33)	41.90 (11.44)		
	MD; 95% CI; *p*	2.60; 0.41 to 4.80; *p* = 0.023 *	9.40; 5.03 to 13.78; *p* < 0.001 *		
Sports	Pre	3.92 (1.97)	3.86 (3.31)	*p* < 0.001 *	0.64
	Post	4.40 (2.16)	6.55 (4.24)		
	MD; 95% CI; *p*	0.48; 0.14 to 0.82; *p* = 0.007 *	2.69; 1.65 to 3.74; *p* < 0.001 *		
QOL	Pre	4.67 (2.59)	4.01 (2.43)	*p* = 0.002 *	0.66
	Post	5.32 (2.88)	7.24 (2.90)		
	MD; 95% CI; *p*	0.65; 0.29 to 1.01; *p* = 0.002 *	3.24; 2.39 to 4.09; *p* < 0.001 *		
NPRS	Pre	6.19 (1.45)	6.48 (1.21)	*p* < 0.001 *	1.05
	Post	4.24 (1.16)	3.21 (0.75)		
	MD; 95% CI; *p*	−1.95; −2.3 to −1.53; *p* < 0.001 *	−3.28; −3.78 to −2.76; *p* < 0.001 *		
Active knee flexion	Pre	112.11 (11.87)	110.65 (9.73)	*p* < 0.001 *	0.61
	Post	115.23 (10.69)	120.17 (4.26)		
	MD; 95% CI; *p*	3.12; 1.56 to 4.68; *p* = 0.001 *	9.53; 5.57 to 13.49; *p* < 0.001 *		
TUG	Pre	9.65 (1.51)	9.46 (0.75)	*p* = 0.001 *	0.47
	Post	9.13 (1.54)	8.58 (0.61)		
	MD; 95% CI; *p*	−0.53; −0.64 to −0.41; *p* < 0.001 *	−0.88; −1.04 to −0.72; *p* < 0.001 *		

Abbreviations: ADL; activities of daily living; HL + EX, high-intensity laser therapy and exercises; KOOS, knee injury and osteoarthritis outcome score; LL + EX, low-level laser therapy and exercises; MD, mean difference; NPRS, numerical pain rating scale; QOL, quality of life; TUG, timed up-and-go test. Note: Values are presented as means and standard deviations. Statistically significant, * *p* < 0.05.

## Data Availability

The datasets analyzed during the current study are available from the corresponding author upon reasonable request. All data from the study are included in this published article.
